# The Candidate Chromosomal Regions Responsible for Milk Yield of Cow: A GWAS Meta-Analysis

**DOI:** 10.3390/ani12050582

**Published:** 2022-02-25

**Authors:** Lida Taherkhani, Mohammad Hossein Banabazi, Nasser EmamJomeh-Kashan, Alireza Noshary, Ikhide Imumorin

**Affiliations:** 1 Department of Animal Science, Science and Research Branch, Islamic Azad University, Tehran 1477893855, Iran; taherkhanilida2121@gmail.com (L.T.); nasser_ejk@yahoo.com (N.E.-K.); 2 Department of Biotechnology, Animal Science Research Institute of Iran (ASRI), Agricultural Research, Education & Extension Organization (AREEO), Karaj 3146618361, Iran; 3 Department of Animal Breeding and Genetics (HGEN), Center for Veterinary Medicine and Animal Science (VHC), Swedish University of Agricultural Sciences (SLU), 75007 Uppsala, Sweden; 4 Department of Animal Science, Karaj Branch, Islamic Azad University, Karaj 3187644511, Iran; alireza.noshary@kiau.ac.ir; 5 School of Biological Sciences, Georgia Institute of Technology, Atlanta, GA 30332, USA; igi2@biology.gatech.edu

**Keywords:** candidate SNPs, dairy cattle, genome-wide association study, meta-analysis, milk yield

## Abstract

**Simple Summary:**

Milk production is one of the most important economic traits in dairy cattle. Therefore, determining the genomic regions influencing this trait can improve milk yield. In this study, we collected data from 16 articles associated with milk yield genome-wide association studies (GWAS) on different cattle breeds. Based on the information from the analysis and level of significance (*p*-value < 2.5 × 10^−6^), we identified different genomic regions on chromosomes with the highest marker density, markers with the highest effect, genes within or near these regions, chromosomes with the greatest effects on milk yield.

**Abstract:**

Milk yield (MY) is highly heritable and an economically important trait in dairy livestock species. To increase power to detect candidate genomic regions for this trait, we carried out a meta-analysis of genome-wide association studies (GWAS). In the present study, we identified 19 studies in PubMed for the meta-analysis. After review of the studies, 16 studies passed the filters for meta-analysis, and the number of chromosomes, detected markers and their positions, number of animals, and *p*-values were extracted from these studies and recorded. The final data set based on 16 GWAS studies had 353,698 cows and 3950 markers and was analyzed using METAL software. Our findings revealed 1712 significant (*p*-value < 2.5 × 10^−6^) genomic loci related to MY, with markers associated with MY found on all autosomes and sex chromosomes and the majority of them found on chromosome 14. Furthermore, gene ontology (GO) annotation was used to explore biological functions of the genes associated with MY; therefore, different regions of this chromosome may be suitable as genomic regions for further research into gene expression.

## 1. Introduction

Milk is an important natural source of nutrients for the growth of newborn mammals. Different methods have been applied to detect genetic factors affecting milk production in dairy cattle, the most recent of which is genome-wide association studies (GWAS). The ultimate goal of GWAS is to identify the dependency between single nucleotide polymorphisms (SNP) and a trait using high-density markers at the genome surface to detect causative mutations that affect the phenotype of a trait [1]. During the last decade, GWAS has become an important source for generating novel hypotheses in the field of genetics. Therefore, GWASs tend to be suitable for detecting common variants associated with specific phenotypes [2].

Using data from GWAS, the meta-analysis technique is used to detect common genomic regions affecting traits by pooling the results of many studies together. Meta-analysis is an essential tool for synthesizing evidence needed to inform clinical decision making and policy. Systematic reviews summarize available literature using specific search parameters followed by critical appraisal and logical synthesis of multiple primary studies [3]. Nowadays, the meta-analysis technique is used in the agricultural and veterinary sciences in order to resolve inconsistencies in the results of scientific sources. Using the meta-analysis technique, which is a systematic and statistical study, data from different studies can be combined to achieve a single conclusion and interpretation. The reason is that individual studies have some limitations regarding the statistical power and reliability of the results. A meta-analysis by combining data and results of different research improves statistical power and accuracy of estimates [4,5]. Meta-analysis is becoming an increasingly important tool in GWAS studies of complex genetic diseases and traits [6]. The aim of this study was to detect the chromosomal regions related to milk yield using meta-analysis of different cow breeds.

## 2. Material and Methods

### 2.1. Data and Literature Review

**Figure 1:**
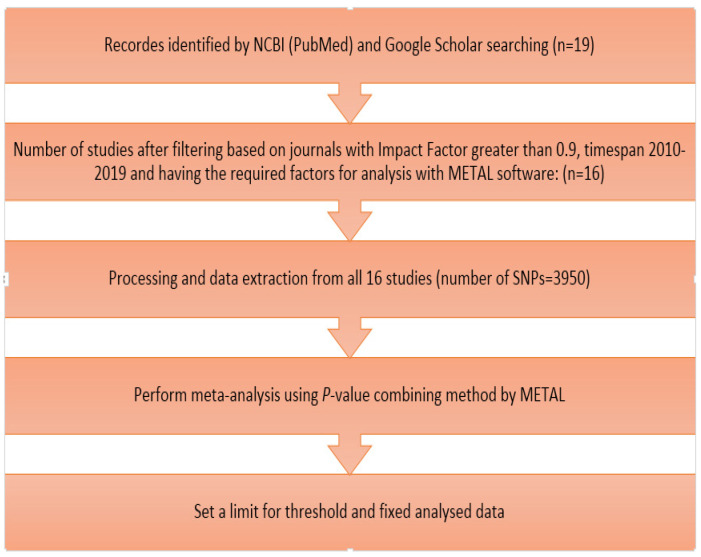
Flowchart of the meta-analysis of milk yield.

The review of GWAS studies on cow milk yield regarding the number of chromosomes and SNP positions reveals details of chromosomal regions that affect the trait. In this study, the data from GWAS tests on MY are from Google Scholar (https://scholar.google.ca/, accessed on 12 December 2019) and the National Center for Biotechnology Information site (www.ncbi.nlm.nih.gov, accessed on 27 December 2019) searched (Figure 1). Using different filters including articles in journals with high impact factors (greater than 0.9) and timespan 2010–2019 and also had the required factors for analysis with METAL software, 16 out of 19 studies were used for meta-analysis. The required information such as marker name and the number of their chromosomes, their position on the chromosome, their *p*-values, and also the number of tested animals of each study was stored in a file. It should be noted that the number of autosomal and sexual SNPs associated with milk yield that were extracted from these 16 articles was 3950.

### 2.2. Meta-Analysis

The meta-analysis was based on the weighted Z-scores model as implemented in the METAL software [7]. It considers the *p*-value, direction of effect, and the number of individuals included in each within-population GWAS study [8].

**Figure 2:**
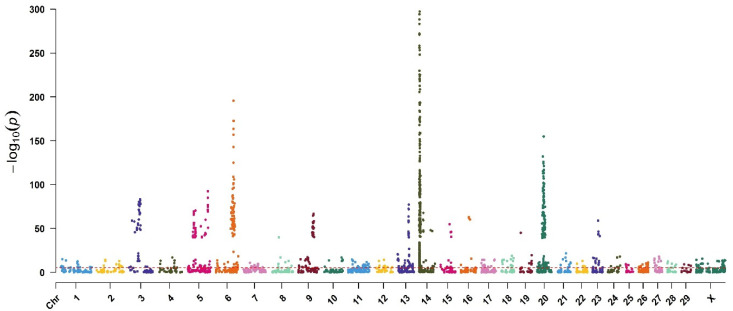
Manhattan plot of the GWAS meta-analysis for milk yield. Red line indicates *p* = 2.5 × 10^−6^.

The GWAS meta-analysis showed the effective chromosomes (Figure 2). For the Manhattan plot, a pre-determined genome-wide significance threshold of 2.5 × 10^−6^ was calculated with formulae 1 and 2 (α = 0.01).

(1) $$x=\frac{\alpha }{\mathrm{NO}.\;\mathrm{SNPs}}$$

(2) −logx=threshold

Using the Ensembl site (http://ftp.ensembl.org/pub/release-103/gtf/bos_taurus/, accessed on 20 August 2020), the calculated data were checked and the loci of the effective markers and the genes were identified.

### 2.3. Downstream Analyses

The genes with variants that were significant in the meta-analysis and detected SNPs located on them were used as input for the gene ontology (GO) test. The GO terms (the significance level < 0.05) enrichment analysis with genes found within the top SNPs was performed. Using GO Consortium (https://biit.cs.ut.ee/gprofiler/gost, accessed on 5 February 2021), to investigate the biological processes of genes associated with MY investigated.

## 3. Results

The number of SNPs affecting the MY with a significance level lower than <2.5 × 10^−6^ were 1712 sites located on all chromosomes and mainly on chromosome 14. The GWAS meta-analysis showed the effective chromosomes by the Manhattan plot (Figure 2). The number of effective SNPs on chromosomes 14, 20, 6, and 5 were 950, 224, 87, and 65, respectively (Table 1). The other 386 identified SNPs with significance levels lower than 2.5 × 10^−6^ were located on the other 26 sex and autosomal chromosomes. The results showed that fifty-five percent of the effective SNPs related to milk yield were located on chromosome 14.

Results for the top loci by *p*-value in the meta-analysis, with the most significant SNP per locus, are presented in (Table 2). The significance level of 1712 identified SNPs in the meta-analysis was compared and 5 SNPs of rs109421300, rs135549651, rs109146371, rs109350371, and BovineHD4100003579 had the smallest *p*-values (Table 2).

The identified SNPs were distributed on 18 genes (regardless of duplicate genes) with the names: DGAT1, ENSBTAG00000015040, RPAP3, ZC3H3, MROH1, MAF1, MAPK15, RHPN1, VPS28, TRAPPC9, ENSBTAT00000065585, ADGRB1, CYHR1, PTK2, PLEC, SCRIB, GML, and FAM135B.

The GO annotation based on biological processes (BP) showed 32 genes involved in biological functions associated with MY. According to the GO term, these candidate genes were found to be enriched in 15 biological processes. All of GO terms for MY-related biological pathways were related to “wound healing”, “metaphase/anaphase transition of meiosis I”, “meiotic chromosome separation”, “cell migration”, “cell motility and locomotion” (Table 3).

The 18 candidate genes for MY resulting from GWAS were associated with the GO terms of *PTK2* (wound healing) also in (response to wounding), *ENSBTAT00000065585* (wound healing, response to wounding, negative regulation of cellular component movement, intermediate filament cytoskeleton organization, intermediate filament-based process, negative regulation of locomotion, negative regulation of cell migration, negative regulation of cell motility), *SCRIB* (wound healing, response to wounding, vesicle targeting, neurotransmitter receptor transport postsynaptic membrane to endosome), *MAPK15* (vesicle targeting, positive regulation of metaphase/anaphase transition of meiosis I, regulation of metaphase/anaphase transition of meiosis I, positive regulation of meiotic chromosome separation, positive regulation of metaphase/anaphase transition of meiotic cell cycle, negative regulation of cellular component movement, negative regulation of locomotion, negative regulation of cell migration, metaphase/anaphase transition of meiosis I, negative regulation of cell motility), *ADGRB1* (negative regulation of cellular component movement, negative regulation of locomotion, negative regulation of cell migration, negative regulation of cell motility), and *PLEC* (wound healing, response to wounding, intermediate filament cytoskeleton organization, intermediate filament-based process).

## 4. Discussion

A genome-wide meta-analysis and enrichment analysis for milk yield was conducted according to the results of 16 studies (on 353,698 cows and 3950 SNPs) from all over the world (Table 4). We confirmed substantial contribution of different chromosomal loci associated with MY in cows. Three of the most important SNPs, i.e., rs109421300, rs135549651, and rs109146371, were located on chromosome 14.

These observations support the notion that the suggestive loci identified in this study, have an outstanding effect on MY. Moreover, fifty-five percent or 995 identified SNPs with a significance level lower than the specified level, were located on chromosome 14. Therefore, it can be concluded that chromosome 14 is the most effective chromosome on MY. The description of its different regions adds to the accuracy of this issue.

The study showed that regions 1,489,496 to 5,494,654 of chromosome 14 had the most effective SNPs compared to other regions of this chromosome. This means that all of the top 45 SNPs on chromosome 14 were located in this region. Only 24 SNPs in this region were located on the genes. Given that, the density of markers in some regions, including 1,675,278 to 1,967,325 and 4,336,714 to 4,468,478, was higher than in other regions, so that 13 SNPs from 45 of them were located on these regions and the most influential SNP (*p*-value: 2.93 × 10^−771^) in this region was on *DGAT1* (Diacylglycerol O-Acyltransferase 1), a protein-coding gene. *DGAT1* is an enzyme that catalyzes the synthesis of triglycerides from diglycerides and acyl-coenzyme A [25]. The *DGAT1* K232A polymorphism was previously shown to have a significant effect on bovine milk production characteristics (milk yield, protein content, fat content, and fatty acid composition) [25]. The next SNP (*p*-value: 1.12 × 10^−710^) was located on the *LOC100141215* gene. Therefore, because these regions have the highest density and the greatest effect, it can be said, the regions with the most impact.

In our study, a gene-set enrichment analysis and a group of GO enriched for MY were related to several traits. More accurate results showed the GO_BP: 0,042,060 (wound healing), GO_BP: 0,009,611 (response to wounding), GO_BP: 0,006,903 (vesicle targeting), GO_BP: 1,905,188 (positive regulation of metaphase/anaphase transition of meiosis I), GO_BP: 1,905,186 (regulation of metaphase/anaphase transition of meiosis I), GO_BP: 1,905,134 (positive regulation of meiotic chromosome separation), GO_BP: 1,902,104 (positive regulation of metaphase/anaphase transition of meiotic cell cycle), GO_BP: 0,098,968 (neurotransmitter receptor transport postsynaptic membrane to endosome), GO_BP: 0,051,271 (negative regulation of cellular component movement), GO_BP: 0,045,104 (intermediate filament cytoskeleton organization), GO_BP: 0,045,103 (intermediate filament-based process), GO_BP: 0,040,013 (negative regulation of locomotion), GO_BP: 0,030,336 (negative regulation of cell migration), GO_BP: 1,990,949 (metaphase/anaphase transition of meiosis I), GO_BP: 2,000,146 (negative regulation of cell motility).

In the continuation of this study, for a better understanding of the mechanisms of MY and the genomic regions involved, it was necessary to analyze the candidate regions obtained from the results of this study. After performing downstream analyses and finding the relation between the identified genes and these terms, we investigated the relation between some of them and MY using studies that have been previously conducted.

Wound healing is a localized process that involves inflammation, wound cell migration and mitosis, neovascularization, and regeneration of the extracellular matrix [26]. Milk of the cow, especially low-fat milk, is a rich source of calcium which can play a significant role in the acceleration of wound healing and increment of healing quality [27]. Calcium has an essential role in wound healing; therefore, healing is known as a calcium-dependent process [27].

The metaphase to anaphase transition is a point of no return; the duplicated sister chromatids segregate to the future daughter cells, and any mistake in this process may be deleterious to progeny [28]. The metaphase to anaphase transition is controlled by a ubiquitin-mediated degradation process [28].

Cell migration is a complex process requiring the coordination of numerous inter- and intracellular events, such as cytoskeleton reorganization, matrix remodeling, cell–cell adhesion modulation, and induction of chemoattractants [29]. Cell migration plays an important role in a variety of normal physiological processes. These include embryogenesis, angiogenesis, wound healing, repairing of intestinal mucosal damage, and immune defense [30]. However, in some pathological conditions, such as atherosclerosis or gastrointestinal ulcers, a large area of denudation is commonly found, and an immediate repair by the reestablishment of the intact monolayer of cells is required [31].

Cell motility is the capacity of cells to translocate onto a solid substratum. This behavior is often a hallmark of fibroblastic cells. In epithelial cells, cell motility occurs after the dissociation of a cell from its neighboring cell(s) and after the modification of its position relative to other cells or a solid substrate [32]. Cell motility plays an integral role in many physiologic and pathologic processes, notably organized wound contraction and fibroblast and vascular endothelial cell migration during wound healing, metastatic tumor cell migration, stem cell mobilization and homing, and tissue remodeling [33]. Sufficient information is not available about other terms and their relation to MY and this requires further investigation.

For a better understanding of the mechanisms of milk production, it is suggested that more downstream analysis on the proposed region affecting MY including pathway analysis is carried out. Furthermore, it may be needed to review the contribution of the genes located in that region on the MY variance. For example, *DGAT1*, which is a major gene for MY, had the highest significant level in this study. Banabazi et al. (2016) have identified SNPs located on the transcribed regions and their 100 K proposed panel performed 2% better than the 700 K panel [34]. It is suggested to check the SNPs located on the candidate region among 1019 loci that they discovered on the transcriptome of chromosome 14 and 24,842 SNPs located on a high-density commercial SNP array (700 K) on the same chromosome. In addition, the comparison between Bos-taurus and Bos-indicus cattle may highlight the importance of the candidate region.

## 5. Conclusions

The most effective SNPs and genes which affect milk yield are located on chromosome 14, and the regions between 1,489,496 to 5,494,654 have the most effective SNPs in terms of the significance level. Emphasis on the use of these SNPs could justify a large part of the genetic variance in MY. Downstream analyses in these regions also partially demonstrated the mechanism of the effect of genes associated with MY in these regions. Additional analysis can help better understand the mechanism of MY in these regions.

## Figures and Tables

**Table 1:** The length of each chromosome and number of effective SNPs on them.

CHR Number	Length (bp)	No. SNPs on CHR
1	158,337,067	15
2	137,060,424	10
3	121,430,405	41
4	120,829,699	6
5	121,191,424	65
6	119,458,736	87
7	112,638,659	12
8	113,384,836	9
9	105,708,250	35
10	104,305,016	6
11	107,310,763	23
12	91,163,125	9
13	84,2403,50	45
14	84,648,390	950
15	85,296,676	13
16	81,724,687	5
17	75,158,596	13
18	66,004,023	14
19	64,057,457	12
20	72,042,655	224
21	71,599,096	10
22	61,435,874	5
23	52,530,062	17
24	62,714,930	16
25	42,904,170	13
26	51,681,464	11
27	45,407,902	11
28	46,312,546	5
29	51,505,224	3
X	148,823,899	34
		1712

**Table 2:** The detailed information of top 50 detected SNPs via meta-analysis in milk yield.

CHR Number	SNP Name	Position	Overlapped Genes	*p*-Value
14	rs109421300	1801116	DGAT1	2.93 × 10^−771^
14	rs135549651	1967325	ENSBTAG00000015040	1.12 × 10^−710^
14	rs109146371	1651311		1.82 × 10^−653^
14	rs109350371	2054457		1.90 × 10^−637^
5	BovineHD410000357	32784231	RPAP3	3.10 × 10^−416^
14	rs109558046	2909929		1.44 × 10^−396^
14	rs109752439	1489496		1.17 × 10^−366^
14	rs110199901	2524432		4.10 × 10^−298^
14	rs110706284	2398876	ZC3H3	6.76 × 10^−295^
14	rs41627764	2276443		5.13 × 10^−289^
14	rs41629750	2002873		6.61 × 10^−284^
14	rs137205809	1892559	MROH1	6.36 × 10^−273^
14	rs137787931	1880378	MROH1	1.44 × 10^−272^
14	rs133119726	1868636	MROH1	9.39 × 10^−272^
14	rs109742607	2217163		4.47 × 10^−259^
14	rs41256919	1923292	MAF1	3.28 × 10^−257^
14	rs110323635	2239085	MAPK15	9.29 × 10^−257^
14	rs109529219	2468020	RHPN1	5.37 × 10^−254^
14	rs110060785	2553525		6.91 × 10^−249^
14	rs17870736	1696470	VPS28	1.86 × 10^−230^
14	rs110892754	2117455		2.82 × 10^−226^
14	rs109086264	4414829	TRAPPC9	7.94 × 10^−226^
14	rs110174651	2754909		3.71 × 10^−224^
14	rs136891853	2764862		2.95 × 10^−221^
14	rs110749653	2138926	ENSBTAT00000065585	3.89 × 10^−221^
14	rs110411273	3640788		1.20 × 10^−219^
14	rs110626984	2674264		8.13 × 10^−219^
14	rs29024688	3297177		2.57 × 10^−213^
14	rs55617160	4468478	TRAPPC9	3.02 × 10^−209^
14	rs134974438	2150825		2.63 × 10^−206^
6	rs110527224	88592295		3.23 × 10^−196^
14	rs110143087	4767039		1.51 × 10^−194^
14	rs109530164	4456595	TRAPPC9	2.40 × 10^−194^
14	rs137757978	2164419		6.16 × 10^−194^
14	rs109225594	4848750		1.00 × 10^−193^
14	rs109545018	3006509	ADGRB1	6.81 × 10^−192^
14	rs109968515	1675278	CYHR1	5.37 × 10^−185^
14	rs110251237	4068825		1.70 × 10^−184^
14	rs110185345	4043743	PTK2	4.90 × 10^−184^
14	rs111018678	4336714	TRAPPC9	7.76 × 10^−178^
14	rs137309662	3371507		2.57 × 10^−176^
14	rs135270011	2084067	PLEC	2.82 × 10^−175^
14	rs108992746	2951045	ADGRB1	7.76 × 10^−174^
6	rs137147462	88887995		2.63 × 10^−173^
6	rs110694875	89139865		2.75 × 10^−173^
14	rs110017379	4364952	TRAPPC9	4.07 × 10^−172^
14	rs41602530	2194228	SCRIB	1.66 × 10^−168^
6	rs42766480	88891318		2.82 × 10^−164^
14	rs719209105	2741434	GML	8.32 × 10^−160^
14	rs110501942	5494654	FAM135B	1.74 × 10^−159^

**Table 3:** Significant biological process associated with genes affecting milk yield.

Term ID	Term Name	*p*-Value (Adj)	Gene Name	Number
GO:0042060	Wound healing	0.033647963	ENSBTAT00000065585, PTK2, PLEC, SCRIB	4
GO:0009611	Response to wounding	0.039101239	ENSBTAT00000065585, PTK2, PLEC, SCRIB	4
GO:0006903	Vesicle targeting	0.048695181	MAPK15, SCRIB	2
GO:1905188	Positive regulation of metaphase/anaphase transition of meiosis I	0.048695181	MAPK15	1
GO:1905186	Regulation of metaphase/anaphase transition of meiosis I	0.048695181	MAPK15	1
GO:1905134	Positive regulation of meiotic chromosome separation	0.048695181	MAPK15	1
GO:1902104	Positive regulation of metaphase/anaphase transition of meiotic cell cycle	0.048695181	MAPK15	1
GO:0098968	Neurotransmitter receptor transport postsynaptic membrane to endosome	0.048695181	SCRIB	1
GO:0051271	Negative regulation of cellular component movement	0.048695181	MAPK15, ENSBTAT00000065585, ADGRB1	3
GO:0045104	Intermediate filament cytoskeleton organization	0.048695181	ENSBTAT00000065585, PLEC	2
GO:0045103	Intermediate filament-based process	0.048695181	ENSBTAT00000065585, PLEC	2
GO:0040013	Negative regulation of locomotion	0.048695181	MAPK15, ENSBTAT00000065585, ADGRB1	3
GO:0030336	Negative regulation of cell migration	0.048695181	MAPK15, ENSBTAT00000065585, ADGRB1	3
GO:1990949	Metaphase/anaphase transition of meiosis I	0.048695181	MAPK15	1
GO:2000146	Negative regulation of cell motility	0.048695181	MAPK15, ENSBTAT00000065585, ADGRB1	3

Note: GO enrichment analysis was performed in candidate genes associated with milk yield (*p*-value < 2.5 × 10^−6^).

**Table 4:** Identified SNPs on each continent. Data extracted from scientific literature published from 2010 to 2019.

Continent	Studies	N ^1^	No. SNPs ^2^	Refs.
Africa	1	250	20	[9]
Asia	5	13,188	74	[10,11,12,13,14]
Europe	5	22,384	1542	[15,16,17,18,19]
North America	4	299,951	2309	[20,21,22,23]
Australia	1	17,925	5	[24]
Global	16	353,698	3950	

^1^ N, number of animals tested; ^2^ no. SNPs, number of detected SNPs on cows in each continent.
